# Biomechanical Consequences of the Elastic Properties of Dental Implant Alloys on the Supporting Bone: Finite Element Analysis

**DOI:** 10.1155/2016/1850401

**Published:** 2016-11-22

**Authors:** Esteban Pérez-Pevida, Aritza Brizuela-Velasco, David Chávarri-Prado, Antonio Jiménez-Garrudo, Fernando Sánchez-Lasheras, Eneko Solaberrieta-Méndez, Markel Diéguez-Pereira, Felipe J. Fernández-González, Borja Dehesa-Ibarra, Francesca Monticelli

**Affiliations:** ^1^Department of Surgery, Gynecology and Obstetrics, Faculty of Sports and Health Sciences, University of Zaragoza, Huesca, Spain; ^2^Department of Stomatology I, Faculty of Medicine and Dentistry, University of the Basque Country, Leioa, Spain; ^3^Department of Surgery, Faculty of Medicine, University of Salamanca, Salamanca, Spain; ^4^Department of Construction and Manufacturing Engineering, Polytechnic School of Engineering, University of Oviedo, Gijon, Spain; ^5^Department of Graphic Expression and Engineering Projects, Faculty of Engineering, University of the Basque Country, Bilbao, Spain; ^6^Faculty of Medicine and Health Sciences, University of Oviedo, Oviedo, Spain; ^7^Department of Orthodontics and Dentofacial Orthopedics, Faculty of Medicine and Health Sciences, University of Oviedo, Oviedo, Spain; ^8^Facultad de Ciencias de la Salud, Universidad Autónoma de Chile, Santiago de Chile, Chile

## Abstract

The objective of the present study is to evaluate how the elastic properties of the fabrication material of dental implants influence peri-implant bone load transfer in terms of the magnitude and distribution of stress and deformation. A three-dimensional (3D) finite element analysis was performed; the model used was a section of mandibular bone with a single implant containing a cemented ceramic-metal crown on a titanium abutment. The following three alloys were compared: rigid (Y-TZP), conventional (Ti-6Al-4V), and hyperelastic (Ti-Nb-Zr). A 150-N static load was tested on the central fossa at 6° relative to the axial axis of the implant. The results showed no differences in the distribution of stress and deformation of the bone for any of the three types of alloys studied, mainly being concentrated at the peri-implant cortical layer. However, there were differences found in the magnitude of the stress transferred to the supporting bone, with the most rigid alloy (Y-TZP) transferring the least stress and deformation to cortical bone. We conclude that there is an effect of the fabrication material of dental implants on the magnitude of the stress and deformation transferred to peri-implant bone.

## 1. **Introduction**

The ability of dental implants to reliably rehabilitate edentulous spaces has been well studied, but these implants are not without their technical and biological problems [1].

One of the more frequent and most important biological issues is marginal crest bone loss around the dental implant. This type of bone loss can be influenced by a number of factors, including infection of the peri-implant tissue, mismatch between the attachment and the implant, surgical trauma, and biomechanical factors related to occlusal load [2]. Wolff's law postulates that bone can be remodeled based on the forces applied during its normal function, modifying its internal and external architecture and changing its shape and density [3, 4]. Mechanically, bone behaves identically to any other material in that it undergoes deformation when subject to a load. In this sense, Frost proposed a criterion for remodeling bone based on the magnitude of the internal stress it undergoes when performing its designated function. In other words, bone can support a set amount of deformation, beyond which microfractures can be produced, which in turn can result in bone loss [5]. Clinically, these microdeformations can translate into micromovements of teeth or implants. In teeth, micromovements are due to the elastic deformation of periodontal ligaments, constituting an unloading of the stress transferred to the support bone; on the other hand, in implants these micromovements are due exclusively to microdeformations of the peri-implant bone. Micromovements greater than 150 *μ*m are not well tolerated by the bone-implant system, potentially translating to a loss of implant osseointegration [6]. In the case of the peri-implant bone, clinical reports describe the loss as occurring at the level of the marginal bone crest [7–9]. This localization coincides with the zones of major stress transfer to the support fixture during the application of functional and parafunctional forces [10].

Compared with the root of a natural tooth, the rigidity of an implant created with a conventional alloy (Ti6-Al-4V) is much greater than the rigidity of the support bone. According to the principle of “composite beam analysis,” when two materials with different elastic moduli (such as bone versus titanium) are placed in contact and one is subject to a load, the greatest stress is localized at the first point of contact between the two materials; in the case of dental implants, this point is the marginal bone crest [11, 12]. Hooke's Law states that the deformation of a material depends on its elastic modulus and the stress it experiences. A greater elastic modulus results in a smaller deformation; thus, in the bone-implant system, it is the bone that tends to suffer greater deformations [13]. In short, to prevent peri-implant marginal bone loss, it is necessary to control the factors that influence the transfer of occlusal load to the bone-implant interface. Chiefly, these factors are the type of load (direction and magnitude), the macroscopic implant design, the implant surface treatment, the quality and amount of peri-implant bone, and the properties of the fabrication material of both the implant and the prosthesis [14].

The most common material used in the fabrication of dental implants is titanium. Traditionally, commercially pure titanium implants are used, but they are limited by the following poor mechanical properties: a relatively lower elastic modulus and tensile strength and a relatively high chance of corrosion. Consequently, there has been a shift to using alloys of titanium with other materials such as vanadium and aluminum instead. These alloys increase the elastic modulus and the tensile strength of the implant while decreasing the chance of corrosion. While the Ti-6Al-4V alloy is the most frequently used in the fabrication of dental implants, new and additional biomimetic alloys are currently being developed to achieve greater biocompatibility and assure correct functioning in the human body [15].

As a result of the demand for smaller implants that can be used in locations with limited bone or prosthetic space availability, more rigid alloys such as Ti-Zr have been developed that can resist potential implant fractures as a result of the application of functional loads [16].

Because of the importance of aesthetics to implant-based prosthetic rehabilitation, there has been a rise in the use of dental implants made with zirconia partially stabilized with yttrium (Y-TZP), producing a more pleasing color than the unaesthetic look of the metal finish of titanium implants [17].

Each of these alloys has a significantly high Young's modulus compared to bone. Young's modulus of cortical bone is 15 GPa with a Poisson ratio of 0.30; in contrast, Young's modulus of the Ti-6Al-4V alloy is 110 GPa, with a Poisson ratio of 0.35, and Young's modulus of the Y-TZP alloy is 210 GPa, with a Poisson ratio of 0.31 [14–17]. As a result, new alloys with elastic properties that better mimic the properties of pristine bone with better biomimetics and biocompatibility than the aforementioned alloys have recently been developed. Chief among these new alloys are hyperelastic alloys, such as titanium-niobium-zircon (Ti-Nb-Zr), which, in addition to titanium and zircon, add metals such as niobium. These additives reduce Young's modulus to 71 GPa, which is closer to that of natural bone [18, 19].

Various studies demonstrate the excellent biomechanical behavior and the biocompatibility of the Ti-Nb-Zr alloy in biomedicine with new thermal alloy and surface treatments, including the addition of new metals such as tantalum [20–22]. Despite these studies, there is no sufficient evidence supporting its use as a fabrication material for dental implants.

In this context, finite element analysis was performed to obtain specific data about both the magnitude and distribution of tension and deformation transferred from the implant to the supporting bone. Numerous articles appear in the literature that have investigated the biomechanical behavior of different types of dental implant and implant-supported prosthetic rehabilitations. But to date no literature has evaluated the biomechanical consequences for the bone supporting the implant, comparing the various alloys used for fabricating dental implants, which present widely varying elasticity.

For this reason, the objective of the present finite element study is to evaluate the influence of the elastic properties of the implant fabrication materials on peri-implant bone load transfer in terms of the magnitude and distribution of stress and deformation.

Our hypothesis is that an implant fabricated using an alloy with relatively low Young's modulus, such as Ti-Nb-Zr, will transfer less stress and produce less microdeformation in the peri-implant bone when compared to alloys with higher elastic moduli.

## 2. **Materials and Methods**

### 2.1. Design of the Finite Element Model

A three-dimensional (3D) finite element model was created to evaluate the magnitude and distribution of the stress in the peri-implant bone of a single implant with a crown cemented to a titanium abutment. The model created was a section of edentulous, posterior mandibular type II bone according to the classification scheme of Lekholm and Zarb [27]. The bone surrounding the implant was 23 mm high and 12 mm wide with a 1-mm-thick cortical bone layer and the rest comprised trabecular bone.

The reference for the macroscopic design of the threaded implant was a standard internal connection implant with the following parameters: a 2.8 mm polished neck (Straumann Standard, Institute Straumann AG, Basel, Switzerland), 10 mm in length, 4.1 mm in body width and 4.8 mm in platform width. The body of the implant was aligned with the treated surface beneath the osseous crest in the cortical bone, simulating the ideal positioning of an implant with these characteristics. The cemented titanium abutment was modeled as a 4.8-mm-wide and 5.5-mm-tall platform (RN synOcta, Institute Straumann AG, Basel, Switzerland) and a titanium retaining screw.

**Figure 1:**
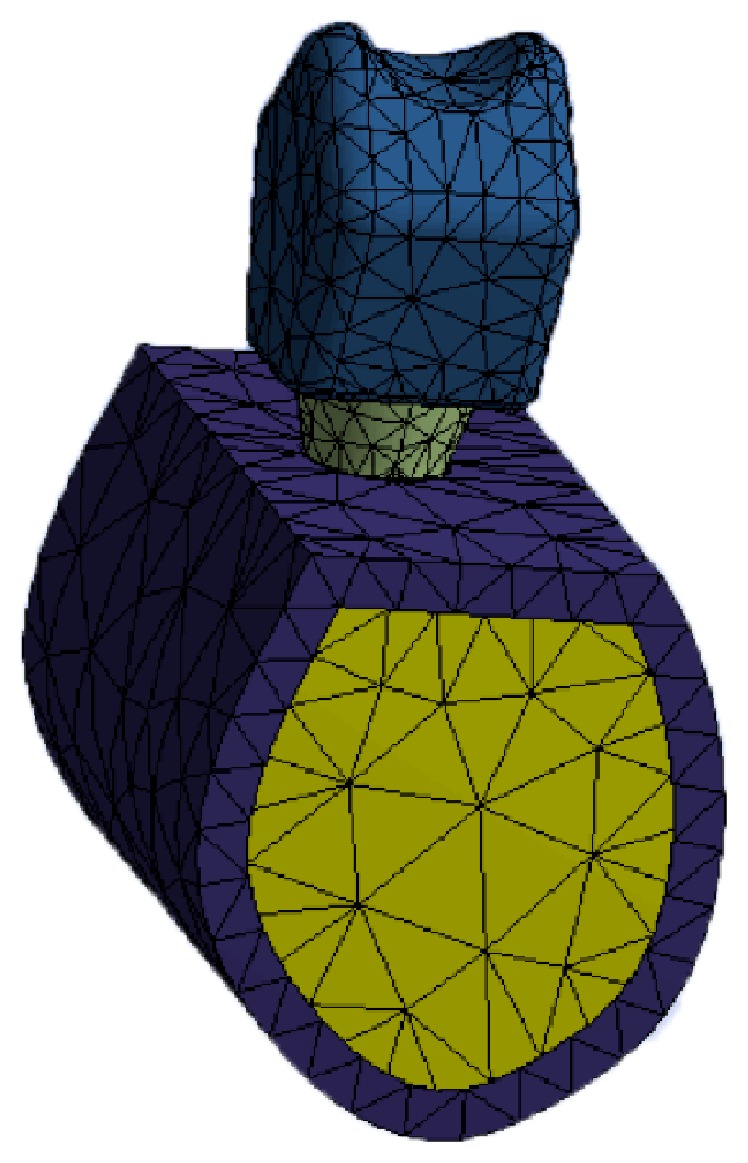
Finite element model used.

A metal-ceramic crown was modeled using a Cr-Co alloy and a feldspathic ceramic surface; the crown was 8 mm tall and 10.6 mm wide, with a thickness of 3 mm (1 mm metal alloy and 1-1 mm ceramic surfacing), and was cemented to the titanium abutment. The finite element model used is shown in Figure 1.

### 2.2. Material Properties and Interface Conditions

The properties of the materials used in the finite element model were obtained from the literature and are listed in Table 1. The materials used in this model are treated as linearly elastic, homogeneous, and isotropic. The interface between the bone and implant is assumed to be a 100% ideal osseointegration. The cement layer between the crown and abutment was ignored, assuming a precise passive fit and an effective joining of the two components. The same model was used for all of the conditions, only changing the appropriate mechanical properties of the implant to compare the behavior of the different fabrication alloys (Ti-6Al-4V, Ti-Zr, Y-TZP, and Ti-Nb-Zr).

### 2.3. Load and Edge Conditions

**Figure 2:**
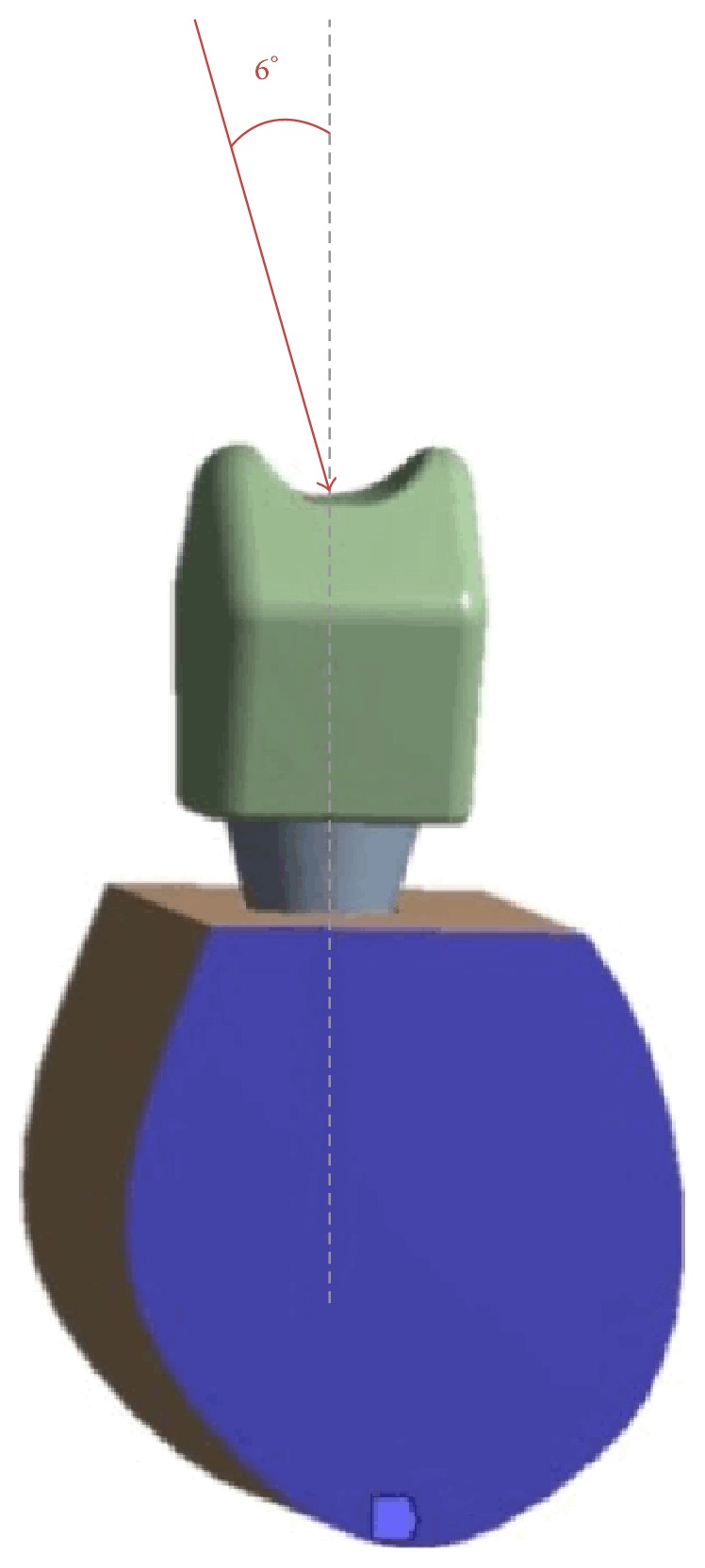
Load conditions used in the finite element analysis.

For each of the conditions, a load of 150 N was applied to the central occlusal fossa of the crown in the buccolingual direction and at 6° relative to the axial axis of the implant as shown in Figure 2, simulating the physiological load conditions of a mandibular premolar-molar section.

Stress (according to the von Mises yield criterion) and deformation data were obtained numerically.

Finite element modeling was performed using the commercial software Ansys 11.0 (Ansys, Swanson Analysis System, Canonsburg, PA, USA). The finite element model used was composed of 33268 elements and 45517 nodes.

## 3. **Results**

The results focus on the highest and lowest von Mises stress values, the stress distribution in the bone surrounding the implant and in the implant itself, and the deformation of both components in the model. To facilitate interpretation of the data, we separate the results for stress and deformation in the cortical bone, in the trabecular bone and in the implant for each of the fabrication alloys.

The maximum and minimum stresses transferred to the bone and implants are shown in Table 2.

In cortical bone, the highest maximum stress transferred was produced in the Ti-Nb-Zr model at 17.271 MPa, while the lowest maximum stress was produced by the Y-TZP model at 16.206 MPa. The opposite holds for the minimum stress transferred; the lowest value was produced by the Ti-Nb-Zr model (0.1416 MPa), while the highest minimum stress was produced by the Y-TZP model (0.1434 MPa). Consequently, the maximum (16.945 MPa) and minimum (0.14238 MPa) stresses delivered by the Ti-6Al-4V alloy were in the middle of these ranges.

The results show that there is greater stress transfer in the cortical bone compared with trabecular bone, independent of the typology of the alloy. Additionally, the results observed in the trabecular bone are opposite of the results observed in the cortical bone: the highest value of maximum stress transferred is caused by Y-TZP with 2.142 MPa, followed by Ti-6Al-4V (2.038 MPa) and Ti-Nb-Zr (1.948 MPa). The values of transferred minimum stress for the trabecular bone were ordered in the same way as the cortical bone; however, the highest value corresponded to Y-TZP (0.03851 MPa), followed by Ti-6Al-4V (0.03779 MPa) and Ti-Nb-Zr (0.03716 MPa).

The results also show that the greatest stress is transferred to the implants, which is significantly different with respect to bone, including the cortical layer. However, although the three models were tested using the same load conditions, the stress imparted is different and is influenced by the elastic properties of the different alloys. In this way, the alloy that received the greatest maximum stress was the most rigid one, Y-TZP, with a value of 113.22 MPa, while the alloy that received the lowest maximum stress was the least elastic alloy, Ti-Nb-Zr, with a value of 76.673 MPa. The value Ti-6Al-4V was consequently between these two values. The same order applies for the minimum transferred stress: the highest value was produced by Y-TZP (95.39 MPa) and the lowest by Ti-Nb-Zr (63.88 MPa).

There were no substantial differences observed in analyzing the stress distributions between the three models. In each case, there is a clear distribution of stress in the most coronal bone region in contact with the implant, which is the cortical bone corresponding to the marginal crest bone. This distribution can be explained using the principle of “composite beam analysis” mentioned previously. The stress transferred to the peri-implant bone is distributed primarily to the side corresponding to the direction of the vector of the applied load. In this case, this vector has a buccolingual direction and so the stress is distributed primarily in the lingual sector of the bone surrounding the implant. There is also some distribution of transferred stress in the bone adjacent to the apex of the implant that corresponds to the axial component of the applied load on the model.

Finally, Table 3 shows the values of the deformation expressed in micrometers (*μ*m). In the cortical bone, the highest value of deformation was observed in the Ti-Nb-Zr alloy (64.99 *μ*m) and the lowest in the Y-TZP alloy (59.97 *μ*m).

Similar results were obtained for the trabecular bone: the highest deformation was found in the Ti-Nb-Zr alloy (62.44 *μ*m) and the lowest in the Y-TZP alloy (58.74 *μ*m).

At the level of the implant itself, the maximum deformation was produced by the Ti-Nb-Zr alloy at 93.97 *μ*m and the lowest by the Y-TZP alloy at 73.09 *μ*m.

## 4. **Discussion**

This study uses a 3D finite element analysis to compare the magnitude and distribution of stress and the deformation of peri-implant bone and the implant itself based on the elastic characteristics of three alloys used in the fabrication of the following dental implants: Y-TZP, Ti-6Al-4V, and Ti-Nb-Zr.

In light of the results observed, it is not possible to completely confirm the hypotheses presented at the start of the study, though differences were observed in the transfer of stress depending on the elastic behavior of the implant. However, these results must be evaluated carefully because validation of the stress analysis using finite element depends on the degree to which material properties and geometries, the applied load, and conditions at the interface align with reality [28]. In this study, it was assumed that the simulated structures in the model were homogeneous, isotropic, and linearly elastic, although these assumptions are not always the case, especially in bone. The assumptions made here, however, taken to simplify the model to be able to complete the analysis, are not different compared to the assumptions made in other studies that evaluate the behavior of stress in models of single implants [12, 23, 24, 29].

Our study used cortical and trabecular bone possessing identical geometries and mechanical properties for each of the models. In this way, the model agrees with a majority of biomechanical studies of finite elements, although there are a number of studies that delineate a transitional bone type with trabecular and cortical properties that is in contact with the surface of the implant and possesses Young's modulus and a Poisson ratio different from the rest of the modeled bone and simulating bone in the process of scarification [30]. In our study, trabecular and cortical bone possess identical mechanical properties in each of the models, given our supposition of established osseointegration versus an ongoing process of bone healing.

Our analysis used an occlusal load of 150 N at an angle of 6° relative to the axial axis of the implant, simulating the average values produced in a patient with dental implants and similar to the normal occlusal forces generated during mastication [31, 32]. During the actual mastication process, however, much more complicated load patterns are produced that are nearly impossible to replicate, necessitating the simplified load conditions used for our models. Not surprisingly, it should be noted that the forces tested in our analysis are essentially static, corresponding to the characteristic forces of a central bruxism, as opposed to masticatory forces, which would be primarily dynamic. Furthermore, the type of load in conjunction with the elastic properties of the support material can influence the biomechanical result. These limitations have to be considered when interpreting the final results.

To better interpret the stress and deformation results, we will concentrate on the Y-TZP and Ti-Nb-Zr materials, as their properties correspond to the extremes of the range of values obtained from our simulations.

**Figure 3:**
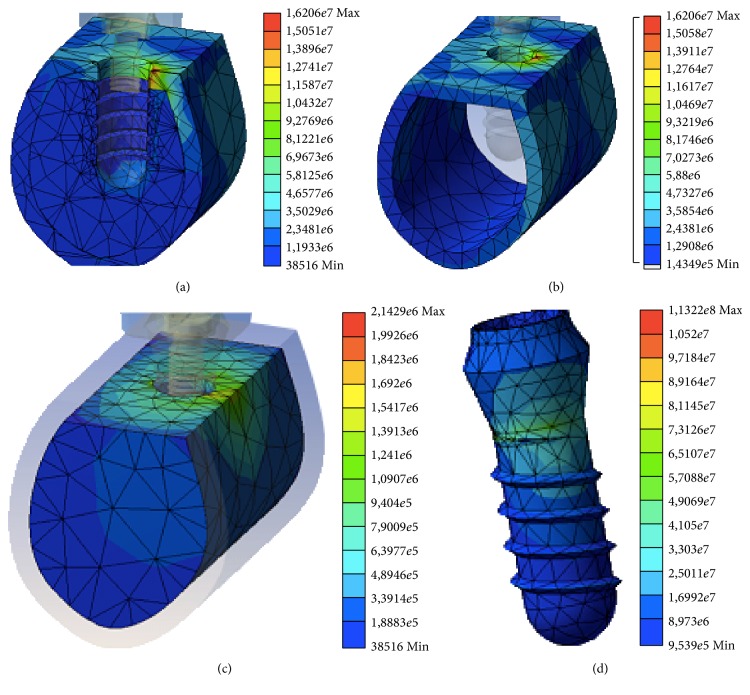
Distribution of the stress in the entire model (a), cortical bone (b), trabecular bone (c), and implant (d) for the Y-TZP material.

**Figure 4:**
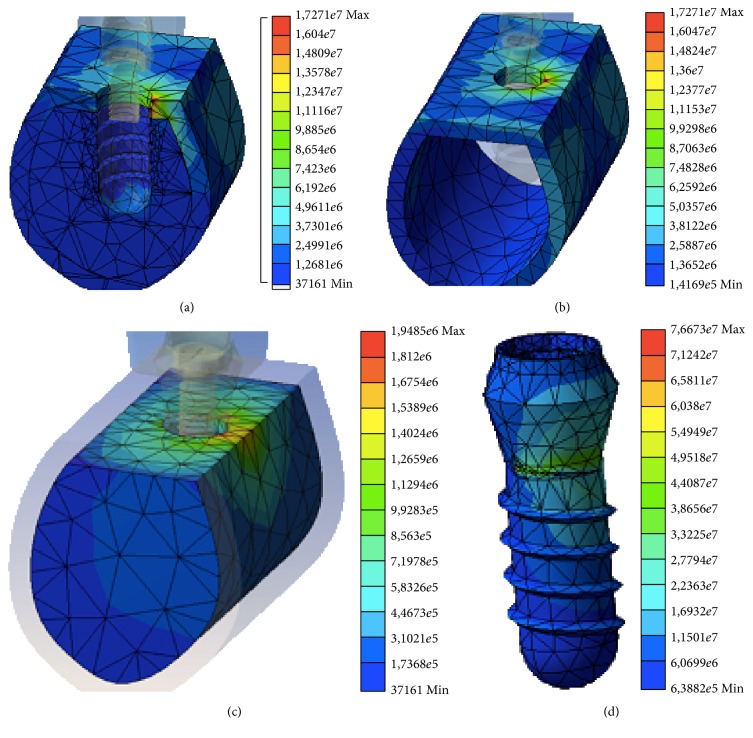
Distribution of the stress in the entire model (a), cortical bone (b), trabecular bone (c), and implant (d) for the Ti-Nb-Zr material.

Following the application of the load, there were no differences observed in the distributions of stress at the surrounding bone due to the different fabrication materials; thus, given the same dental implant design, the mechanical properties of the fabrication material do not seem to affect the distribution of stresses in the peri-implant bone. Figures 3 and 4 show the stress distributions in the bone, both cortical and trabecular, and in the implants created from Y-TZP (Figure 3) and Ti-Nb-Zr (Figure 4). In both cases, the peaks of stress for all the variables were located in the marginal cortical bone in contact with the implant on the side corresponding to the directional vector of the applied load. These results are in line with the majority of studies using finite element testing for single implants [12, 23, 24, 29].

Similarly, there were no differences observed in the distribution of the stress transferred to the implant across the different fabrication materials used; however, there were significant differences in the magnitudes of the stress delivered to each of the different implants. The material that received the greatest stress was Y-TZP, while the one that received the least amount of stress was Ti-Nb-Zr; thus, there appears to be a direct relationship between Young's modulus of the material and the stress transferred to the implant itself. This result can be explained by the elastic characteristics of the fabrication material; for the same load and implant design, a more rigid implant absorbs more stress. Similar results were obtained by Çaglar et al. in their analysis of finite element comparing zircon and titanium implants [33–35]. These results also correspond to the results of Osman et al. who analyzed a denture model and, in comparing the two materials, found similar results but with smaller differences in the two materials [36], likely because the design of the prosthesis was different from the one studied here and from the designs in the previously cited studies.

The greatest von Mises stress transferred to the cortical bone was produced around the Ti-Nb-Zr implant, which was the one with the smallest elastic modulus. Thus, an inverse relationship between the elastic modulus of the implant and the stress transferred to the cortical bone seems to exist.

The opposite result occurred in the trabecular bone: the lowest stress occurred around the implant created from Ti-Nb-Zr. Therefore, the alloy with the lowest elasticity modulus appears to transfer less stress to the bone structure with a lower Young's modulus and thus is closer to the implant material; yet it transfers the greatest amount of stress to cortical bone, which has a greater elastic modulus.

It can therefore be argued that when the peri-implant bone possesses a greater Young's modulus, for example, that of cortical bone, better biomechanical behavior and therefore lower stress transfer to bone are achieved with implant fabrication materials with high elastic moduli, such as the Y-TZP alloy used in our study. On the other hand, for bone with a lower elasticity modulus, such as trabecular bone, less stress is transferred when the implant is made using alloys with mechanical properties similar to bone; therefore, the ideal implant material would have a low Young's modulus, such as the Ti-Nb-Zr alloy in our model. Çaglar et al. evaluate the biomechanical behavior of zircon and titanium implants and obtain results similar to ours: the stress transfer to cortical bone is greater with implants with lower Young's moduli. However, they observe similar results when looking at the stress at the trabecular bone, while our analysis shows that less stress is transferred using an implant with a low Young's modulus, albeit with smaller differences than the differences found in cortical bone [33].

The cortical bone thus absorbs the greater part of the distribution of transferred stress. To prevent this bone from being subjected to even greater stress, implants made from alloys with a high elastic modulus could have better biomechanical behavior. Additionally, in implants, the marginal cortical bone is of vital importance to the maintenance and correct prognosis of implant-based prosthetic rehabilitation, and thus, it is important that the distribution of transferred stress here be supported by the structure.

In both bone structures (cortical and trabecular), we found an inverse relationship between deformation and Young's modulus of the fabrication material; that is, when the elastic modulus is low, the deformation experienced by both the cortical and trabecular bone is high. The deformation data obtained in the cortical and trabecular bone for the same fabrication material are very similar, meaning that although cortical bone receives more stress than trabecular bone, they deform practically the same amount due to the higher elastic modulus and rigidity of the cortical bone.

This behavior of stress distribution is in line with the basic principle of the conservation of energy; for the same load, the implant that receives less stress transfers more at the first point of contact with bone and thus transfers less to the rest of the bone. That is, an implant with a low elastic modulus (Ti-Nb-Zr) absorbs less stress but transfers more stress to cortical bone and less to trabecular bone and vice versa for the implant with a high elastic modulus (Y-TZP).

The deformation that the implant undergoes is also inversely proportional to Young's modulus of its fabrication material. The implant that underwent the greatest deformation was the Ti-Nb-Zr implant, which concurs with the increase in deformation of the bone surrounding the implant because maintaining good osseointegration between bone and implant requires the bone to deform just as much as the implant does.

Extrapolating the deformation results for peri-implant bone in this in vitro study to clinical reality, the deformations obtained are so low that they have to be measured in micrometers, the largest produced where cortical bone made contact with the Ti-Nb-Zr implant (64.99 *μ*m). These deformation values could be compatible with a good prognosis of implant-based prosthetic rehabilitation because they do not pass the 150 *μ*m threshold, the accepted tolerance limit of the system. Deformations that exceed this amount could translate to loss of implant osseointegration [6]. However, importance should be given to the quantitative results of this study; the finite element model created here is carried out using a perfect physiological load on the implant, which are load conditions that are difficult to replicate in vivo.

Given the limitations of extrapolating results to clinical practice and the fact that it is impossible to reproduce oral physiological and anatomical conditions exactly in finite elements analysis, the present results should be treated with caution.

Nevertheless, according to the results, implant with a high elastic modulus would appear to display better biomechanical behavior, particularly when in contact with cortical bone with a higher elastic modulus, in which the greater percentage of tension is distributed prior to functional loading.

## 5. Conclusions

On the basis of the data analysis and given the limitations of the finite element analysis, we can conclude the following:

1. The dental implant fabrication material affects the magnitude of the stress transferred both to the peri-implant bone and to the implant itself.
2. The greatest transferred stress was obtained from cortical bone using a Ti-Nb-Zr implant. In bone with a high Young's modulus or in cortical bone, the greatest stress transfer occurs when the fabrication material of the implant has a low Young's modulus.
3. There were no significant differences among the three implant fabrication materials with regard to the distribution of stress in either the surrounding bone or the implant itself. The stress is distributed primarily in the marginal crest region of the peri-implant cortical bone.
4. There is a proportional, inverse relationship between the deformation of the peri-implant bone and the dental implant fabrication material. A low elastic modulus in the fabrication material results in greater cortical and trabecular bone deformation, contrary to our initial hypothesis.

## Figures and Tables

**Table 1:** Mechanical properties of materials and fixtures.

Material	Component	Young's modulus (GPa)	Poisson ratio	Reference
Cortical bone		15	0.30	Geng et al. [23]
Spongy bone		1	0.25	Geng et al. [23]
Y-TZP	Implant	210	0.31	Piconi and Maccauro [17]
Ti-6Al-4V alloy	Abutment and screw	107.2	0.30	Álvarez et al. [24]
	Implant	110	0.35	Álvarez et al. [24]
Ti-Nb-Zr alloy	Implant	71	0.32	López et al. [25]
Cr-Co alloy	Crown interior	218	0.33	Álvarez et al. [24]
Feldspathic porcelain	Crown surface	65	0.25	Bona et al. [26]

**Table 2:** Maximum and minimum von Mises stresses (MPa) in cortical and trabecular bones and implants for all fabrication materials.

Fabrication material		von Mises stress (MPa)		
		Cortical	Trabecular	Implant
Y-TZP	Min	0.1434	0.03851	0.953
	Max	16.206	2.142	113.22
Ti-6Al-4V	Min	0.14238	0.03779	0.748
	Max	16.945	2.038	91.23
Ti-Nb-Zr	Min	0.1416	0.03716	0.638
	Max	17.271	1.948	76.673

**Table 3:** Maximum and minimum deformations (*μ*m) in cortical and trabecular bone and in implants for the different fabrication materials.

Fabrication materials		Deformation (*μ*m)		
		Cortical	Trabecular	Implant
Y-TZP	Min	0	0	45.711
	Max	59.971	58.745	73.093
Ti-6Al-4V	Min	0	0	45.006
	Max	62.516	60.55	83.145
Ti-Nb-Zr	Min	0	0	44.492
	Max	64.999	62.441	93.979
